# Plasma ALS and Gal-3BP differentiate early from advanced liver fibrosis in MASLD patients

**DOI:** 10.1186/s40364-024-00583-z

**Published:** 2024-04-29

**Authors:** David Pérez Compte, Lucas Etourneau, Anne-Marie Hesse, Alexandra Kraut, Justine Barthelon, Nathalie Sturm, Hélène Borges, Salomé Biennier, Marie Courçon, Marc de Saint Loup, Victoria Mignot, Charlotte Costentin, Thomas Burger, Yohann Couté, Christophe Bruley, Thomas Decaens, Michel Jaquinod, Jérôme Boursier, Virginie Brun

**Affiliations:** 1 Univ. Grenoble Alpes, INSERM, CEA, UA13 BGE, CNRS, FR2048 ProFI, EDyP team, 17 Avenue des Martyrs, 38000 Grenoble, France; 2 Université Grenoble Alpes, CNRS, UMR 5525, VetAgro Sup, Grenoble INP, TIMC, 38000 Grenoble, France; 3 Université Grenoble Alpes, Clinique Universitaire d’Hépato-Gastroentérologie, CHU Grenoble Alpes, 38000 Grenoble, France; 4 Univ. Grenoble Alpes, Institute for Advanced Biosciences-INSERM U1209/ CNRS UMR 5309, Grenoble, France; 5 Hepato-Gastroenterology Department, University Hospital, Angers, France; 6 HIFIH Laboratory, UPRES 3859, SFR 4208, LUNAM University, Angers, France; 7 Univ. Grenoble Alpes, CEA, Leti, 38000 Grenoble, France

**Keywords:** Biomarker, MASLD, Fibrosis, Proteomics, Liver

## Abstract

**Background:**

Metabolic dysfunction-associated steatotic liver disease (MASLD) is estimated to affect 30% of the world’s population, and its prevalence is increasing in line with obesity. Liver fibrosis is closely related to mortality, making it the most important clinical parameter for MASLD. It is currently assessed by liver biopsy – an invasive procedure that has some limitations. There is thus an urgent need for a reliable non-invasive means to diagnose earlier MASLD stages.

**Methods:**

A discovery study was performed on 158 plasma samples from histologically-characterised MASLD patients using mass spectrometry (MS)-based quantitative proteomics. Differentially abundant proteins were selected for verification by ELISA in the same cohort. They were subsequently validated in an independent MASLD cohort (*n* = 200).

**Results:**

From the 72 proteins differentially abundant between patients with early (F0-2) and advanced fibrosis (F3-4), we selected Insulin-like growth factor-binding protein complex acid labile subunit (ALS) and Galectin-3-binding protein (Gal-3BP) for further study. In our validation cohort, AUROCs with 95% CIs of 0.744 [0.673 – 0.816] and 0.735 [0.661 – 0.81] were obtained for ALS and Gal-3BP, respectively. Combining ALS and Gal-3BP improved the assessment of advanced liver fibrosis, giving an AUROC of 0.796 [0.731. 0.862]. The {ALS; Gal-3BP} model surpassed classic fibrosis panels in predicting advanced liver fibrosis.

**Conclusions:**

Further investigations with complementary cohorts will be needed to confirm the usefulness of ALS and Gal-3BP individually and in combination with other biomarkers for diagnosis of liver fibrosis. With the availability of ELISA assays, these findings could be rapidly clinically translated, providing direct benefits for patients.

**Graphical Abstract:**

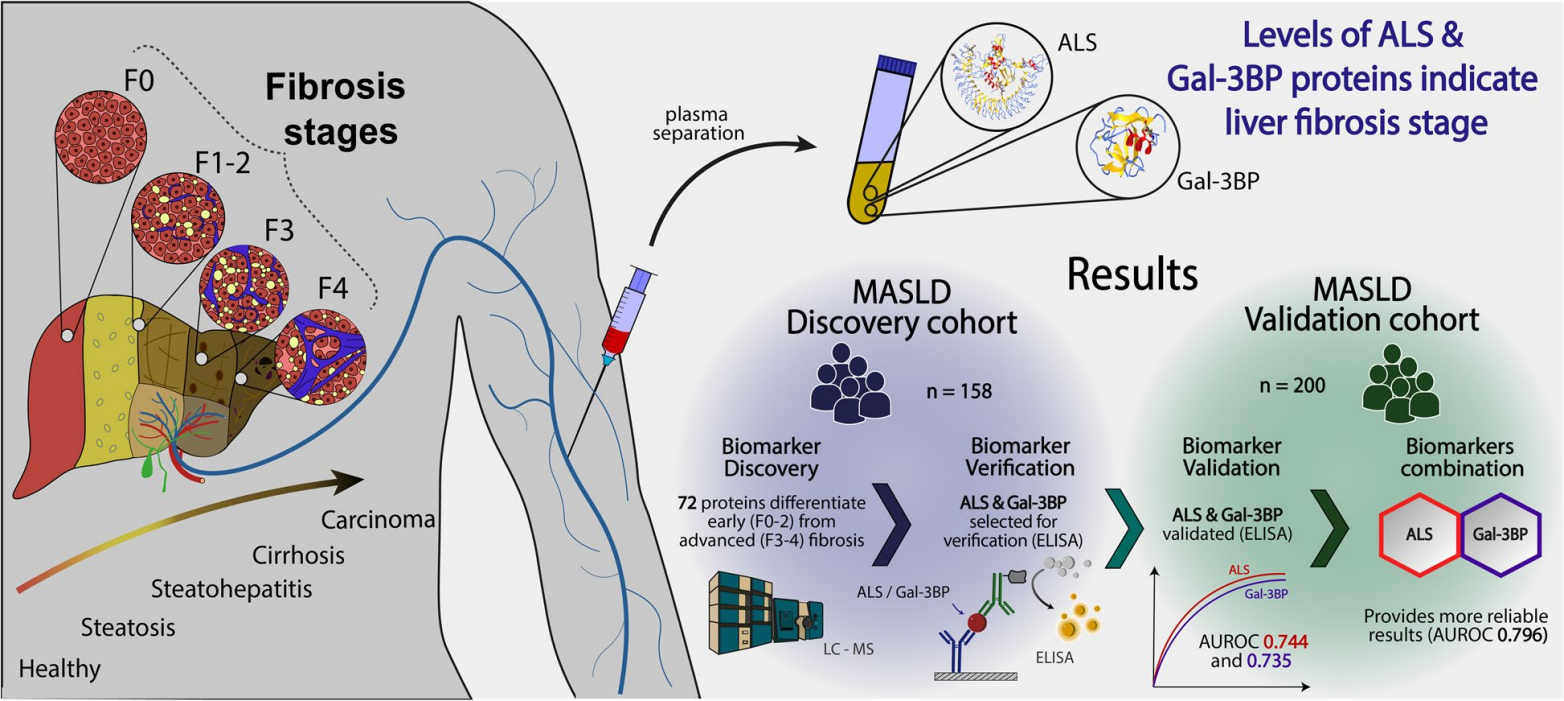

**Supplementary Information:**

The online version contains supplementary material available at 10.1186/s40364-024-00583-z.

## Background

Liver diseases are a major global health problem, accounting for more than 2 million deaths worldwide each year [1]. Linked to the spread of obesity, Metabolic dysfunction-associated steatotic liver disease (MASLD) has become an epidemic affecting 1.66 billion adults in 2019, and its prevalence is increasing [1–3]. This disease has several stages, starting from steatosis and progressing to metabolic dysfunction-associated steatohepatitis (MASH), cirrhosis, or hepatocellular carcinoma (HCC) [4]. The early stages may be asymptomatic, but progress can be rapid. In the most severe cases, liver transplantation (LT) is required [5, 6]. MASLD is the fastest-growing indication for LT in Western Countries [7]. Although LT is the only curative treatment for patients with advanced MASLD, it remains a major intervention that can only be performed on patients who meet strict eligibility criteria, and when donor livers are available. We lack effective pharmacological options for MASLD, although numerous clinical trials of potential treatments are in advanced phase II or III [8–10]. Lifestyle modifications can stop disease progression, and sometimes even reverse the damage on the condition that the disease is caught in its early stages [11].

The most reliable method available to diagnose and characterise MASLD is liver biopsy, which can be used to stratify patients with liver steatosis, fibrosis, or inflammation [12]. However, liver biopsy remains expensive and invasive, it also provides suboptimal performance due to sampling variability and low histological reproducibility [13]. Given that more than a quarter of the world’s population has MASLD, a reliable and cost-effective non-invasive screening test would greatly simplify and accelerate early diagnosis for those concerned. Over the past 15 years, several techniques have been developed as potential substitutes for liver biopsy [13]. However, because MASLD involves many molecular mechanisms that can lead to a range of pathophysiological states, no replacement for biopsy has yet been found that provides an equivalent level of information to clinicians. Nevertheless, some tests can guide medical decisions if they specifically target critical parameters and have acceptable performance levels.

Based on its link with mortality [14], fibrosis is the most urgent variable to be determined in MASLD, and an essential parameter for the medical decision-making process. Liver biopsies are therefore considered when there is a significant risk of advanced liver fibrosis [8]. Clinicians have defined a histological score to stratify fibrosis development from stage F0 (no fibrosis) to stage F4 (cirrhosis) included in a composite SAF (Steatosis, Activity and Fibrosis) score [15]. The distinction between early stages (F0 to F2) and advanced stages (F3 to F4) is critical, as it determines patient management [16]. However, liver biopsy is not without risks, and presents a certain number of limitations [13]. The search for non-invasive fibrosis tests has therefore driven intense efforts. In this regard, several simple composite scores such as the FibroTest [17], the MASLD Fibrosis score [18] or the enhanced liver fibrosis (ELF) test [19] have been developed. More recently, scores based on multiple parameters and including more complex algorithms incorporating MASLD-related protein biomarkers have been proposed [20, 21]. However, although their performances are close to that of liver biopsy, none has reached the level required to replace it, and the urgent need for alternative non-invasive biomarkers remains [22].

Over the past two decades, due to continuous progress in automation, instrumentation and data processing, mass spectrometry (MS)-based proteomics has become a remarkably efficient means to characterize biofluid proteomes in various pathophysiological contexts [23]. As a result, it is now possible to characterise large cohorts of patients in a reasonable timeframe to identify biomarker candidates [24, 25]. Here, an unbiased MS-based discovery study to identify plasma biomarkers was conducted on samples collected from histologically-characterised patients with suspected MASLD (*n* = 158). Two candidate biomarkers were selected, and their discriminatory performance verified using ELISA assays. Results from a validation study performed on an independent cohort (*n* = 200) compared favourably with those from widely used fibrosis diagnosis methods, such as Fib-4 [26], NFS [18], FibroQ [27] or FibroTest [17].

## Methods

### Patient cohorts

The discovery and verification studies were conducted using samples from a cohort of patients with suspected MASLD (*n* = 158; 94 male; 64 female) recruited at Grenoble Hospital, France. The validation study was performed on samples from an independent cohort of patients with suspected MASLD (*n* = 200; 135 male; 65 female) constituted at Angers Hospital, France. Inclusion criteria for patients were suspicion of MASLD at a clinical consultation and suspicion of advanced fibrosis based on clinical chemistry analysis and imaging exploration. If patients met these criteria, a liver biopsy and histological examination were performed to confirm the diagnosis (Table 1) (details about liver biopsy examination in Supp. Mat. 1). Patients were excluded in case of concomitant treatments with steatosis-inducing drugs (such as corticosteroids, tamoxifen, amiodarone, or methotrexate), excessive alcohol consumption (> 30 g/day in men or > 20 g/day in women), chronic liver diseases (e.g., hepatitis B or C infection), history of liver-related complications (ascites, variceal bleeding, jaundice, encephalopathy, hepatocellular carcinoma), liver biopsy length < 10 mm, VCTE failure, or missing blood markers as input for fibrosis tests. All patients were attending hepatology clinics and no biopsies were performed during bariatric surgery.

**Table 1:** Clinical, biological, and histological characteristics of patients in the Grenoble and Angers cohorts (*n*=158 and *n*=200, respectively)

Variable	Grenoble 158	Angers 200	*p*-values
Age (year)	55 ± 12	57 ± 12	0.029*
Weight (kg)	89 ± 18	94 ± 19	0.023*
Height (cm)	169 ± 10	167 ± 10	0.231
Waist size (cm)	108 ± 15	113 ± 14	0.0054*
Body Mass Index (BMI)(Kg/m^2^)	30.9 ± 5.5	33.4 ± 6.1	0.00014*
Biological and clinical features			
Haptoglobin (g/L)	1.25 ± 0.61	1.24 ± 0.55	0.859
A2M (g/L)	2.25 ± 0.87	2.06 ± 0.82	0.027*
ApoA1 (g/L)	1.46 ± 0.25	1.39 ± 0.21	0.029*
Albumin (g/L)	44.8 ± 4.0	42.4 ± 3.7	9.00E-10*
CRP (mg/L)	4.3 ± 5.5	4.3 ± 4.9	0.677
ALT UI/L	68.4 ± 34.3	63.7 ± 41.9	0.030*
AST UI/L	41.2 ± 21.6	45.5 ± 26.3	0.099
GGT UI/L	153 ± 187	117 ± 170	0.186
Total cholesterol (g/L)	1.96 ± 0.54	1.86 ± 0.45	0.061
HDL cholesterol (g/L)	0.53 ± 0.47	0.43 ± 0.11	0.0076*
LDL cholesterol (g/L)	1.16 ± 0.43	1.20 ± 0.42	0.328
Triglycerides (g/L)	1.77 ± 1.22	1.70 ± 1.12	0.976
Urea (g/L)	5.4 ± 1.5	5.6 ± 2.0	0.603
Bilirubin (µmol/L)	11.2 ± 6.1	12.8 ± 7.2	0.028*
Histological features			
Steatosis grade, n (%)			
0	10 (6.3%)	15 (7.5%)	0.3759
1	31 (19.6%)	91 (45.5%)	
2	54 (34.2%)	63 (31.5%)	
3	63 (39.9%)	31 (15.5%)	
Activity			
Hepatocellular ballooning, n (%)			
0	29 (18.3%)	37 (18.5%)	0.2311
1	72 (45.6%)	83 (41.5%)	
2	57 (36.1%)	80 (40.0%)	
Lobular inflammation, n (%)			
0	49 (31.0%)	37 (18.5%)	0.8138
1	84 (53.2%)	136 (67.5%)	
2	25 (15.8%)	27 (13.5%)	
Fibrosis, n (%)			
0	24 (15.2%)	22 (11.0%)	0.777
1	30 (19.0%)	49 (24.5%)	
2	46 (29.1%)	54 (27.0%)	
3	33 (20.9%)	61 (30.5%)	
4	25 (15.8%)	14 (7.0%)	
MASH, n (%)	102 (64.6%)	148 (74.0%)	0.03809*

BMI calculated as Weight/Height^2^ (kg/m^2^). *A2M* Alpha-2 macroglobulin, *ApoA1* Apolipoprotein A1, *ALT* and *AST* Alanine and aspartate aminotransferases, *GGT* Gamma-glutamyltransferase, *CRP* C-reactive protein, *LDL *and *HDL* Low-density and high-density lipoprotein. The disease activity was calculated by adding the histological score for hepatocellular ballooning to the score for lobular inflammation. The variable MASH corresponds to patients with a score of at least 1 for both steatosis and disease activity (ballooning + lobular inflammation) *P*-values to determine the statistical significance of variable distribution across both cohorts were calculated using a Mann-Whitney test, applying a threshold of 5% (*). *P*-values to determine the statistical significance of the proportion of patients for each hepatic state across both cohorts was verified using a Chi-squared test, applying a threshold of 5% (*)

To cope with potential confounders, distributions for variables from Table 1 comparing early (F0-2) and advanced (F3-4) fibrosis groups within each cohort were evaluated (Supp. Table 3). Significant differences were found for the following variables: age, A2m, AST, ALT, GGT. For variables A2m, AST, ALT and GGT, the differences were expected as these variables are known to be linked to patients’ liver status.

We also assessed the relative proportions of patients presenting a particular histological stage (steatosis, lobular inflammation, bloating and fibrosis). To determine the statistical significance of any differences, we used a Chi-squared test. According to the results obtained, for most histological conditions, the proportions were not significantly different, except for the proportion of patients with MASH, for which a p-value of 0.03809 was determined. Therefore, our two patient cohorts are similarly distributed in terms of liver disease, but with differences in the distribution of certain variables. Consequently, if the combination of biomarkers is relevant for both cohorts, it is an indication of robustness of our panel.

### Sample preparation – in-solution tryptic digestion

Blood from patients was collected within minutes/hours of the biopsy in anticoagulant (EDTA)-treated tubes. Samples were then centrifuged to isolate plasma and stored at -80 °C. Plasma samples were collected between 2013 and 2021. For protein analysis, samples were randomly distributed using Well Plate Maker [28] and then proteins were denatured, reduced, alkylated, and digested with Trypsin/Lys-C Mix before MS-based proteomic analysis (detailed protocols in Supp. Mat. 2 and 3).

#### MS-based proteomic analyses

Briefly, plasma samples were analysed by nano-liquid chromatography (LC) coupled to MS/MS. MS and MS/MS data were acquired in data-dependent acquisition mode (for further information, see Supp. Mat. 4).

#### MS-based proteomics data processing

Data were processed automatically using Mascot Distiller software (version 2.7.1.0, Matrix Science). Peptides and proteins were identified using Mascot (version 2.6) through concomitant searches against Swiss-Prot (*Homo sapiens* taxonomy, downloaded in November 2019), a classical contaminants database (homemade), and their corresponding reversed databases. Trypsin/P was chosen as enzyme, and a maximum of two missed cleavages was allowed. Precursor and fragment mass error tolerances were set to 10 ppm and 25 mmu, respectively. Peptide modifications allowed during the search were: (1) cysteine carbamidomethylation (fixed); (2) acetylation of the protein’s N-terminus (variable), and (3) methionine oxidation (variable). Proline software (version 2.1) [29] was used to merge data for all patients. After merging, results were filtered: conserving rank 1 peptide-spectrum matches with a minimal length of 7 amino acids and a minimal Mascot peptide score of 25. With these parameters, the False Discovery Rate (FDR) for peptide/spectrum match identifications was below 1% according to the target-decoy approach. A minimum of two peptides was required for a protein group to be identified. Proline was then used to perform MS1-based label-free quantification of the protein groups identified, based on their specific peptide abundances (Supp. Table 1).

#### Statistical analysis of MS-based quantitative proteomics data

Data were statistically analysed using Prostar (version 1.26.4) [30]. Protein sets were filtered out if they were not quantified across 70% of patient samples. Proteins identified in the contaminant database were discarded. After log2 transformation, protein abundances were normalised by applying the variance stabilising normalisation method [31]. Missing values were imputed using the Structured Least Square Adaptative (SLSA) method [32]. The statistical significance of any distinction between early fibrosis (F0-2) and advanced fibrosis (F3-4) was verified using the Limma test [33]. Differentially abundant proteins were selected using a p-value cut-off providing a FDR of less than 5% according to the Benjamini–Hochberg procedure (Supp. Table 1) [34].

#### ELISA procedure (Supp. Mat. 5)

Commercial ELISA kits were used to quantify ALS and Gal-3BP in plasma in the verification (Grenoble cohort) and validation (Angers cohort) studies. Tests were performed according to the manufacturer’s instructions, as detailed in Supp. Mat. 5. Full ELISA results can be found in Supp. Table 2.

#### FibroTest-inspired panels

To develop the panel, the ELISA concentrations (ng/mL) of ALS and Gal-3BP variables measured in the validation cohort were combined in a logistic regression model with the objective of obtaining a score differentiating patients with early fibrosis from those with advanced fibrosis. The Grenoble cohort was used as the learning set and the Angers cohort as the test set. AUROC analyses, including calculation of CIs, were performed to compare the different conditions. Further details can be found in Supp. Mat. 6 and 7.

## Results

### Identification of liver fibrosis biomarkers by MS-based discovery proteomics

MS-based label-free quantitative proteomics was used on 158 plasma samples (Grenoble cohort) from patients with suspected MASLD. The aim was to identify candidate biomarkers whose abundance levels can differentiate early fibrosis (F0-2) from advanced fibrosis (F3-4). The strategy deployed reliably identified and quantified 235 plasma proteins, detected in at least 70% of the samples.

**Fig. 1:**
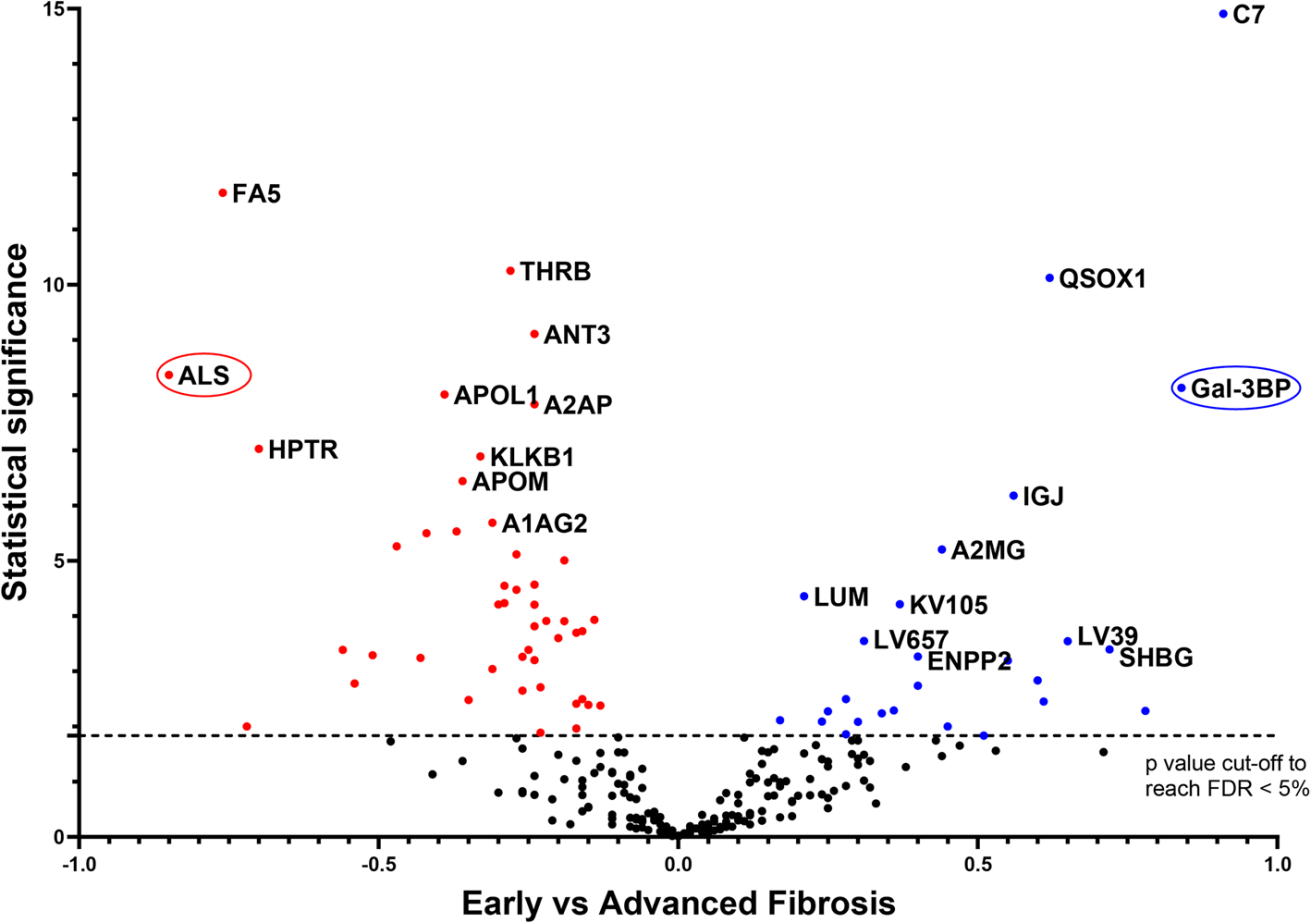
Differentially-abundant proteins identified in plasma from patients with suspected MASLD with early (F0-2) or advanced (F3-4) liver fibrosis. Plasma samples were collected from patients with suspected MASLD and early or advanced fibrosis, as determined from liver biopsies. Plasma proteins were submitted to MS-based label-free quantitative proteomics. For each protein, the Volcano plot displays the –log_10_(Limma p-value) on the Y-axis; the X-axis corresponds to the log_2_ fold-change between early and advanced fibrosis. The cut-off for statistical significance was set for a p-value providing an FDR of less than 5% according to the Benjamini–Hochberg procedure. Red and blue dots represent less abundant and more abundant proteins, respectively, when advanced stages are compared to early stages. Only the 10 most statistically significant proteins in each condition are annotated in this figure (for the complete list, see Supp. Table 1)

Statistical analysis (Limma test) of the difference between the relative abundances of each protein in samples from patients with early or advanced fibrosis revealed 72 differentially abundant proteins (FDR below 5% according to the Benjamini–Hochberg procedure, Fig. 1). Among them, 26 were more abundant and 46 were less abundant in plasma from patients with advanced fibrosis compared to patients with early fibrosis (Supp. Table 1).

### Verification and validation of biomarkers

**Fig. 2:**
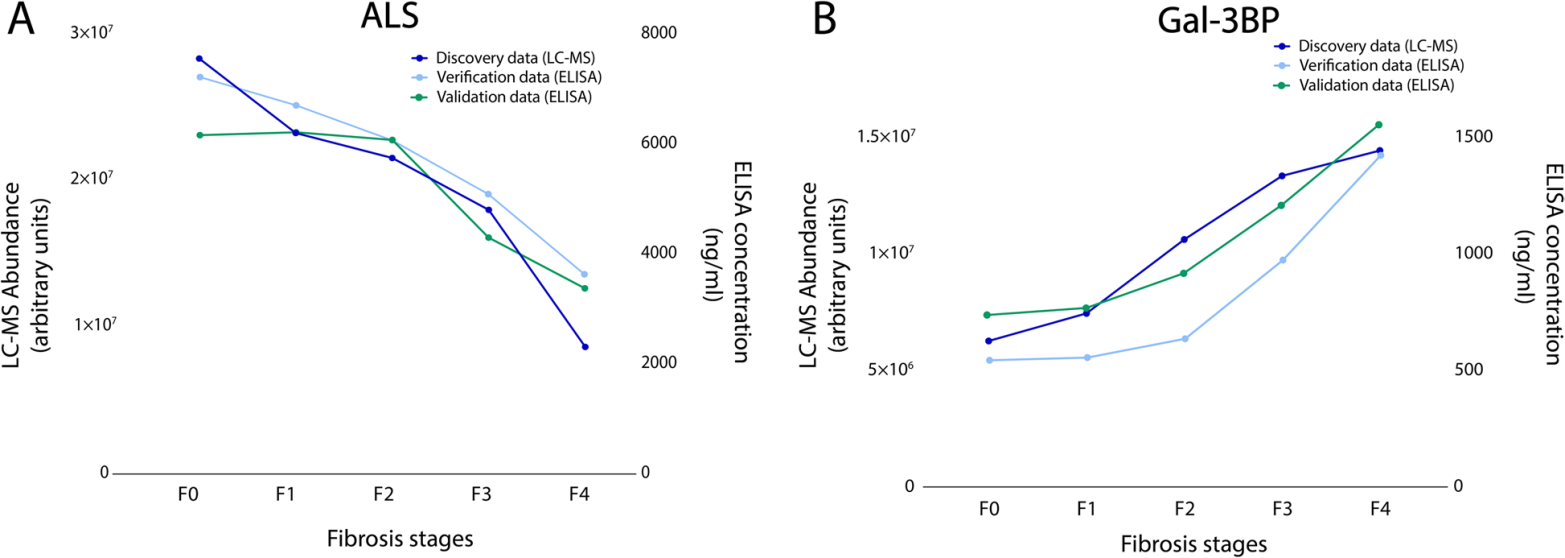
Mean abundances of ALS and Gal-3BP in plasma samples correlate with fibrosis stages and phases. ALS (**A**) and Gal-3BP (**B**) levels based on MS abundance (discovery study, dark blue, left Y-axis, arbitrary units) and concentrations based on ELISA measurements (verification and validation studies, light blue and green, right Y-axis ng/mL)

In the verification phase, immunoassays were used to confirm that the abundance levels of specific proteins differed significantly between samples from the advanced and early fibrosis groups. Candidates for verification were selected if the discovery proteomics results indicated a substantial fold-change between the conditions compared. Of note, C7 was not included in the selection since ELISA assay showed insufficient quantification performance. Two proteins were selected for the verification study: ALS and Gal-3BP. Their concentrations were determined by ELISA in the same 158 plasma samples from the Grenoble cohort. The results showed similar trends in terms of relative abundances for each protein between the discovery and verification studies for the different fibrosis stages (Fig. 2A and B).

Afterwards, the validation study was conducted to confirm the usefulness of the biomarkers with samples from an independent cohort. For this study, 200 samples from patients with suspected MASLD attending Angers Hospital were analysed by ELISA. Importantly, the trends for ALS and Gal-3BP abundances correlated with fibrosis stages in a similar way for the discovery/verification (Grenoble) and validation (Angers) cohorts, whatever the analytical method used (Fig. 2A and B).

**Fig. 3:**
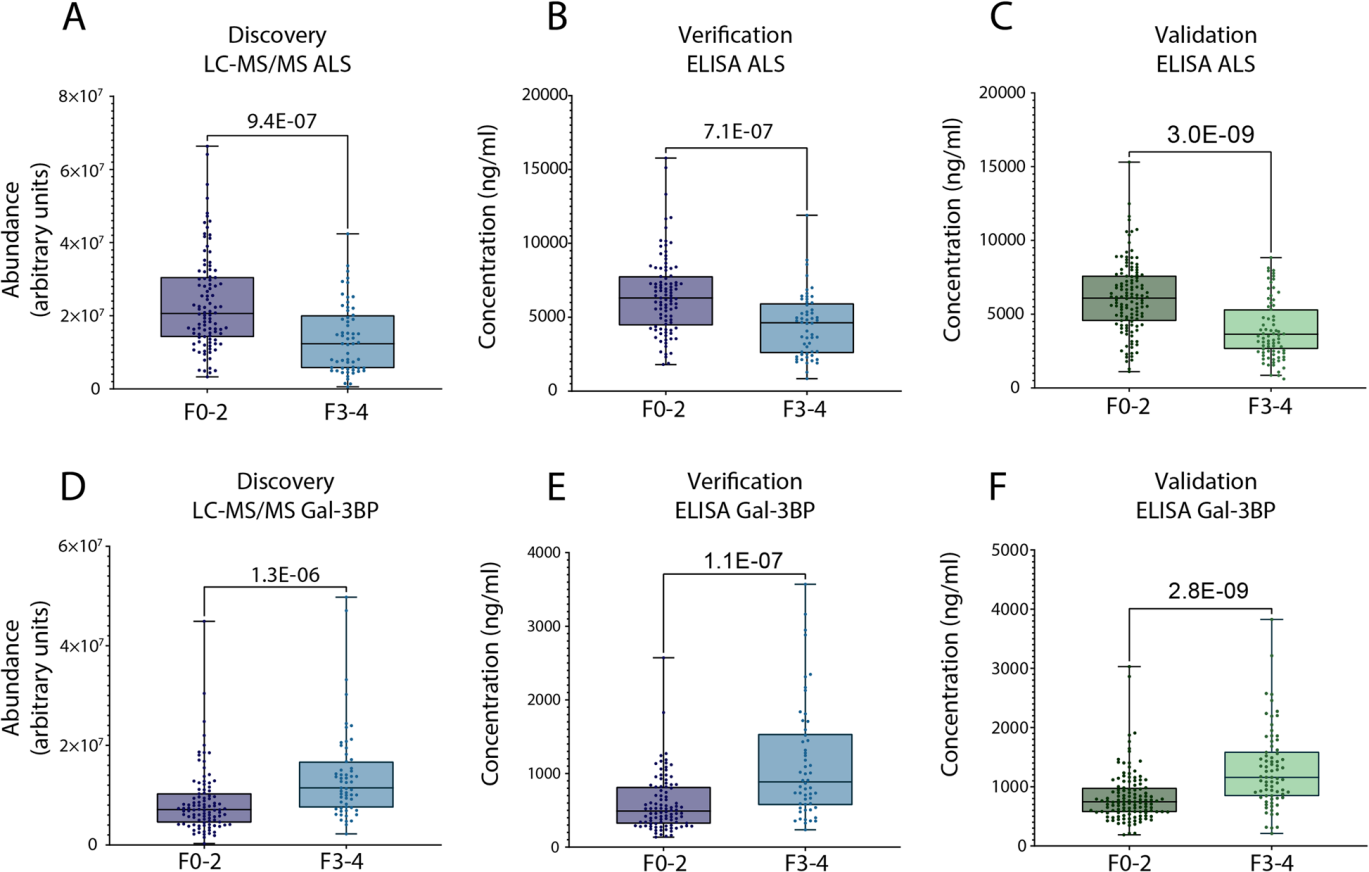
ALS and Gal-3BP abundances can distinguish between patients with early and advanced fibrosis. ALS abundance in discovery (MS analysis, arbitrary units) and verification (ELISA, concentration) studies – Grenoble cohort (**A** and **B**) and in validation study – Angers cohort (ELISA, concentration, **C**). Gal-3BP abundance results from discovery/verification tests – Grenoble cohort (**D** and **E**) and validation test – Angers cohort (**F**). Data are represented as boxplots. Statistical significance was verified using Mann–Whitney. Dots represent ALS and Gal-3BP abundance for individual patients

Finally, differences between early (F0-2) and advanced stages (F3-4) of fibrosis in the discovery/verification and validation cohorts were assessed to determine their statistical significance by applying a Mann–Whitney test (Fig. 3). Statistically significant differences were obtained for the discovery, verification and validation studies for ALS (9.4E-07, 4.4E-07, 3.0E-09, respectively. Figure 3.A, B and C) and Gal-3BP (1.3E-06, 4.1E-07, 2.8E-09, respectively. Figure 3.D, E and F). These results strongly suggest that the abundance of both proteins may discriminate patients with suspected MASLD with early liver fibrosis from those with advanced liver fibrosis.

### Comparison with non-invasive methods for the assessment of liver fibrosis

**Fig. 4:**
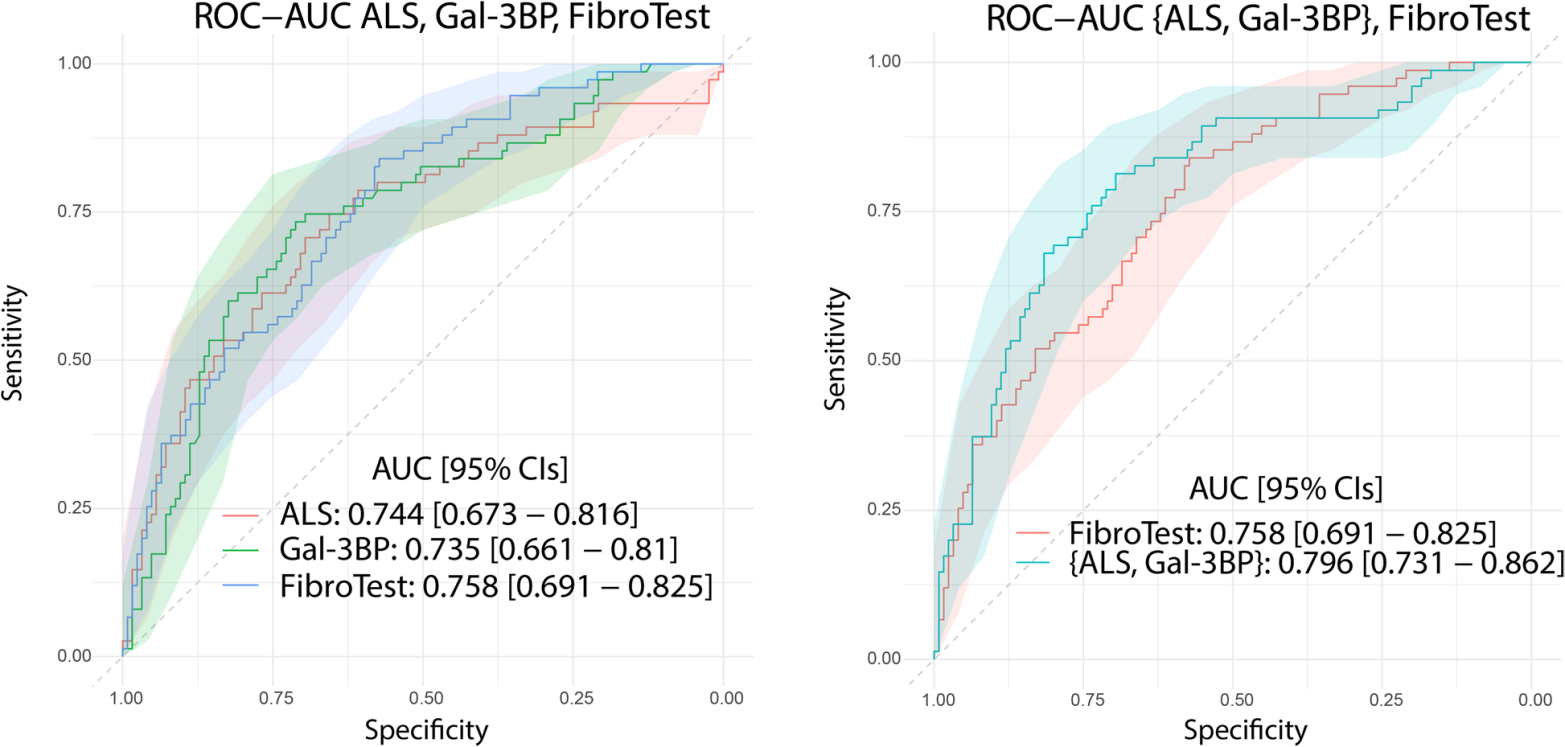
Plasma concentration of ALS and Gal-3BP discriminate early (F0-2) from advanced (F3-4) fibrosis as well as the FibroTest panel; as a 2-protein panel they outperform FibroTest. ROC curves and AUROCs are shown with their respective 95% CIs for ALS/Gal-3BP quantified by ELISA, along with original FibroTest score (left). Combined concentrations of ALS and Gal-3BP compared to original FibroTest (right). Data presented correspond to the validation cohort. Details about CI calculation can be found in Supp. Mat. 6; the curves are displayed on two distinct plots for the sake of clarity only, to avoid confusion due to the extensive overlaps in CIs

We then set out to determine to what extent ALS and Gal-3BP could differentiate early from advanced liver fibrosis, evaluating the biomarkers first independently and then in combination. For this analysis, we performed an AUROC calculation on the validation cohort (Angers) using (1) the ALS concentration measured by ELISA, (2) the Gal-3BP concentration measured by ELISA, and (3) the combined concentrations of Gal-3BP and ALS (Fig. 4). The AUROC values were first compared to those obtained with the FibroTest (for the same cohort), which is widely used and has been tested in several MASLD studies [35, 36]. FibroTest was initially defined as a panel of three protein concentrations, one enzymatic activity, one metabolite concentration, age and sex, combined in a logistic regression model and validated on independent cohorts [17]. In our comparison, ALS and Gal-3BP provided surprisingly similar performances to the original FibroTest: ALS 0.744 [0.673 – 0.816], Gal-3BP 0.735 [0.661 – 0.81], FibroTest 0.758 [0.691 – 0.825] (Table 2). Notably, their 95% CIs were found to largely overlap (Fig. 4). Using the same logistic regression methodology applied to the discovery cohort (Grenoble), we then determined the weightings for ALS and Gal-3BP as a two-protein panel, {ALS, Gal-3BP}, from the curves presented in Fig. 4. With an AUROC of 0.796 [0.731 – 0.862], the {ALS, Gal-3BP} panel performed better for fibrosis differentiation as compared to ALS or Gal-3BP alone, but also compared to the FibroTest panel. Details on sensitivity, specificity, positive predictive values (PPV) and negative predictive values (NPV) for ALS and Gal-3BP proteins, as well as the {ALS; Gal-3BP} combination, can be found in Supp. Table 4.

**Table 2:** AUROCs and 95%CIs for ALS, Gal-3BP, {ALS; Gal-3BP} and classic fibrosis panels and scores used as first and second line tests

Variable	AUROC [95% CIs]
Proteomics biomarkers	
ALS	0.744 [0.673 – 0.816]
Gal-3BP	0.735 [0.661 – 0.810]
{ALS; Gal-3BP}	0.796 [0.731 – 0.862]
Second line tests	
FibroTest	0.758 [0.691 – 0.825]
Fibroscan	0.856 [0.804 – 0.909]
First line tests	
APRI	0.696 [0.621 – 0.771]
LOK Index	0.677 [0.599 – 0.755]
NFS	0.676 [0.602 – 0.751]
FIB-4	0.641 [0.608 – 0.762]
FibroQ	0.631 [0.551 – 0.710]
Forns Index	0.624 [0.542 – 0.706]
AAR	0.581 [0.500 – 0.663]
HUI score	0.577 [0. 494 – 0.660]

Finally, we compared the performance of ALS, Gal-3BP and their combination with other first- and second-line tests (Table 2). The AUROCs obtained were calculated on the validation cohort. The ALS, the Gal-3BP and the {ALS; Gal-3BP} models performed better than all the other tests except for the Fibroscan.

## Discussion

MASLD is a global health problem because of its prevalence and its consequences. We currently lack validated therapies, and reliable non-invasive early diagnostic methods. Fibrosis remains the most important parameter to clarify, allowing clinicians to assess liver-related mortality, and to adapt their prescription to provide more effective treatment [14, 37]. It is therefore urgent to identify biomarkers that can differentiate early (F0-2) from advanced (F3-4) stages of fibrosis to better monitor the disease.

To this end, we designed a discovery proteomics study to detect variations of individual protein abundances in the plasma of patients with suspected MASLD. Among the proteins identified as differentially abundant in plasma from MASLD patients with different stages of liver fibrosis, ALS and Gal-3BP were selected for further investigation. This selection was made based on their abundance difference between fibrosis conditions and the availability of effective commercial ELISA assays. Their validation in an independent cohort of 200 patients using different proteomic approaches (LC–MS and ELISA) is one of the major strengths of this study.

These two proteins are involved in biological mechanisms linked to the pathophysiological processes behind MASLD, such as insulin regulation [38], activation of the immune system [39], or interactions with the extracellular matrix [39]. Decreased ALS levels have previously been associated with the progression of fibrosis in patients with hepatitis C virus [40] or with alcoholic hepatitis [41]. Following two proteomics discovery studies, a decrease in ALS plasma abundance was reported in MASLD patients with advanced fibrosis [42, 43]. Interestingly, proteins from the Insulin growth factor (IGF) family have also been identified as potential biomarkers of fibrosis in patients with MASLD: IGF-1 (Insulin-like Growth Factor 1) [44] and IGFBP3 and 4 (Insulin-like Growth Factor Binding Protein-3 and -4) [44, 45]. For Gal-3BP, other authors have already linked it to MASLD and fibrosis using immunostaining techniques [46], LC–MS proteomics approaches [42], or aptamers [47, 48]. Gal-3BP levels have also been shown to increase with liver fibrosis stages in hepatitis C patients [49]. Ongoing research is focused on the development of anti-fibrotic treatments targeting galectin-3, the ligand of Gal-3BP [50].

Many panels have already been developed in attempts to better stratify MASLD patients according to the different stages of liver fibrosis [17, 18, 51]. Some of the most recent panel developments are based on proteomics studies using aptamers. For example, a panel of 8 proteins including ADAMTSL2 designed to differentiate F0-1 from F2-4 in MASLD patients obtained an AUROC of 0.87 and 0.89 in validation cohorts [52], and the FIBC3 panel, detecting advanced fibrosis stages (F ≥ 3), obtained an AUROC of 0.83 [53]. Our results are comparable, but have the advantage of presenting a panel that can be promptly introduced into clinical practice thanks to the availability of ELISA assays. Indeed, one current limitation of aptamer-based assays is their implementation as viable diagnostic tools, in the clinical environment. Here, we demonstrated that the combination of ALS and Gal-3BP (0.796 [0.731 – 0.862]) significantly improved the diagnosis of advanced fibrosis in our validation cohort compared to the FibroTest panel. Furthermore, the results obtained with the {ALS; Gal-3BP} model proved to be better than those obtained with most conventional tests for the discrimination of early and advanced liver fibrosis (Table 2). Comparison with the ELF test, which demonstrates good accuracy in discriminating between early and advanced fibrosis [54–56] was not possible as serum PIIINP and TIMP-1 dosage were not available in the two cohorts. Overall, despite the variability in the clinical, biological, and histological characteristics of the patients included in the cohorts studied here (Table 1), ALS, Gal-3BP and {ALS; Gal-3BP} effectively differentiated MASLD patients with early fibrosis from those with advanced fibrosis in both cohorts. This suggests a strong potential for generalisation to other cohorts. These results now need to be validated in additional independent cohorts to confirm the clinical utility of these biomarkers.

Although our study was performed on two independent cohorts and the results were concordant using two distinct analysis methods, it does have its limitations. First, the stage of fibrosis was determined by liver biopsy, which was only performed on patients with suspicion of MASLD and advanced fibrosis. Indeed, the selection of patients for liver biopsy is often based on clinical criteria such as elevated liver enzymes, serum markers of fibrosis or other non-invasive tests for liver fibrosis. These criteria can introduce a bias in the composition of the cohort by primarily including patients with advanced disease or, in some cases, patients without steatosis. In the discovery and validation cohorts, 6.3% and 7.5% of patients, respectively, did not have steatosis, it is important to keep these patients in the analysis as they meet all the criteria established by clinicians for suspected advanced liver fibrosis in the context of MASLD. In addition, the biomarkers and panels developed could provide clinicians with additional information about the likelihood of the patient having advanced fibrosis and potentially avoid the need to perform biopsies in patients with early or no fibrosis. Second, because histological lesions are unevenly distributed throughout the liver parenchyma, sampling errors in liver biopsies may lead to misdiagnosis and staging for MASLD patients, as reported elsewhere [13]. Liver biopsy results may also be more or less accurate depending on the length of the sample [57], the distribution of histological lesions in the liver [58], or inter- and intra-observer variability [13]. These parameters could lead to significant misdiagnosis and erroneous staging of MASLD. Finally, the fact that the two distinct quantitative techniques used here – MS and ELISA – provided equivalent results in terms of performance lends support to the validity of the biomarkers identified, while also eliminating any risk of instrumental bias. The advantage of ELISA over LC–MS when analysing large cohorts is that it can be performed in a shorter timeframe and that it is more suitable for use in a hospital setting or clinical laboratory environment. In contrast, LC–MS analysis requires tight instrumental control due to its inherent complexity and sensitivity to technical variations [59].

## Conclusion

In summary, our MS-based proteomic discovery study applied to plasma samples identified numerous proteins that could differentiate the stages of liver fibrosis in patients with suspected MASLD. Among them, two proteins (ALS and Gal-3BP) were of particular interest. Their respective performances were verified with a second analytical technique and validated using an independent cohort of samples (*n* = 200). The combination of ALS and Gal-3BP provided an AUROC of 0.796 [0.731—0.862], exceeding the results of other non-invasive fibrosis assessment models. In view of these promising results, further investigations are now needed on complementary cohorts. Longitudinal studies could also be performed to better understand the behaviour of these proteins as fibrosis progresses in individual patients.

### Supplementary Information

**Supplementary Material 1.**

**Supplementary Material 2.**

## Data Availability

The mass spectrometry proteomics data have been deposited to the ProteomeXchange Consortium via the PRIDE partner repository (https://www.proteomexchange.org/) under dataset identifier PXD043340. Username: reviewer_pxd043340@ebi.ac.uk Password: jM8845UU.
