# Supplementary material for: Plasma ALS and Gal-3BP differentiate early from advanced liver fibrosis in MASLD patients
Source: Biomark Res. 2024 Apr 29;12:44. doi: 10.1186/s40364-024-00583-z (PMC11057169; doi:10.1186/s40364-024-00583-z)
Supplement: Supplementary file 1 — Supplementary Material 1. [file 40364_2024_583_MOESM1_ESM.docx]

Supplementary Material:

1. *Liver Biopsy examination:*

Data on liver biopsy were collected when available at baseline. Liver histological lesions were evaluated in each centre by a senior expert specialized in [hepatology](https://www.sciencedirect.com/topics/medicine-and-dentistry/hepatology) as per the NASH CRN classification^1^. Inter-observer agreement for liver fibrosis staging in MASLD has been shown to be very good to excellent between expert [pathologists](https://www.sciencedirect.com/topics/medicine-and-dentistry/pathologist) from tertiary centers^1–3^. Non-alcoholic steatohepatitis (MASH) was defined as the presence of all 3 of the following conditions: [steatosis](https://www.sciencedirect.com/topics/medicine-and-dentistry/steatosis) grade ≥1, lobular inflammation grade ≥1, and ballooning grade ≥1. Fibrosis was staged as follows: F0 = no fibrosis, F1 = perisinusoidal or portal/periportal fibrosis, F2 = perisinusoidal and portal/periportal fibrosis, F3 = bridging fibrosis and F4 = [cirrhosis](https://www.sciencedirect.com/topics/medicine-and-dentistry/liver-cirrhosis). “Advanced fibrosis” was defined as fibrosis stage F≥3, and “no/mild fibrosis” as F0–2.

1. *Sample preparation – in-solution tryptic digestion.*

All chemicals, reagents, and organic solvents were purchased at the highest grade available from Sigma-Aldrich (St. Louis, MO, U.S.A.). Plasma samples were distributed into 96-well plates, with positions defined by Well-Plate Maker (WPM). Each well contained 3 µL of plasma. Samples were denatured, reduced and alkylated in a single step using a guanidinium chloride (Gnd-HCl) / Tris(2-carboxyethyl)phosphine (TCEP) / chloroacetamide (CAA) mix (final concentrations 2 M / 10 mM / 40 mM in 100 mM ammonium bicarbonate pH 8.2, final volume 20 µL) and incubated at 95 °C for 5 min (in a PCR thermocycler with heated lid). Samples were then digested in a two-step protocol with 3 µg Trypsin/Lys-C mix (Mass Spec Grade, Promega, Madison, WI, U.S.A) for one hour at 37 °C (dilution of Gnd-HCl to 0.5 M) followed by addition of a further 3 µg Trypsin/Lys-C mix for overnight digestion at 37 °C (dilution of Gnd-HCl to 0.2 M). Digested peptides were desalted on a Strata-X Microelution 96-Well SPE Plate, 2 mg/well (Phenomenex, Torrance, CA, U.S.A) in line with the vendor's instructions, and aliquoted into four recovery plates before vacuum-drying (detailed protocol in Supp. Mat. 3). Ultrapure water was prepared with a Merck Millipore Milli-Q® unit (Reference A+ Darmstadt, Germany).

1. *Detailed protocol for sample preparation for LC-MSMS discovery proteomics:*

Mix 1:

- Add 1760 µL ammonium bicarbonate (500 mM)
- Add 880 µL TCEP (100 mM)
- Add 704 µL CAA (500 mM)
- Add 2200 µL Gnd-HCl (8 M)
- Add 1936 µL Ultrapure water

Mix 2:

- Add 660 µL Trypsin/Lys-C mix 1 µg/µL (From 7 Trypsin/Lys-C source tubes resuspended in 100 µL buffer each)
- Add 12.54 mL ammonium bicarbonate (100 mM)

Mix 3:

- Add 660 µL Trypsin/Lys-C mix 1 µg/µL
- Add 25.74 mL Ammonium bicarbonate (100 mM)

In each well:

- Add 3 µL of patient plasma according to the WPM
- Add 17 µL Mix 1 (to each well)
- Heat for 5 min at 95 °C in the PCR machine with plugs
- Add 60 µL Mix 2 (to each well)
- Heat for 1 h at 37 °C in a humidified oven
- Add 120 µL Mix 3 (to each well)
- Heat overnight at 37 °C in a humidified oven

Desalting on Strata-X Microelution 96-Well SPE Plate, 2 mg/well:

- Add 200 µL of methanol to each well
- Add 200 µL of milliQ water to each well
- Add sample
- Wash with 200 µL 5% methanol
- Elute with 2 x 50 µL 2% formic acid in methanol (wait 1 min and aspirate into new plate)
- Aliquot immediately into 4 x 20 µL in 4 snap-off plates
- Leave residue in the elution plate
- Dry using Speed Vac®
- Freeze -20 °C

Resuspension for injection:

- Add 34 µL of 20% acetonitrile, 0.5% trifluoroacetic acid to each well
- Mix well by pipetting around 10 times
- Prepare 10X HRM-IRT® (Biognosys, Schlieren, Switzerland) as recommended by manufacturer
- Dilute to 0.667 UI/µL in water: 4 µL 10X HRM-IRT + 56 µL water
- Vial: 2 µL sample + 8 µL diluted HRM-IRT
- Mix in insert and centrifuge
- Inject 1.5 µL of the final solution

1. *MS-based proteomics analyses:*

An aliquot of each sample was resuspended in 170 µL of 4% acetonitrile, 0.1% trifluoroacetic acid containing HRM-IRT solution. Of this mixture, 1.5 µL (around 500 ng material) was analysed by online nanoLC–MS/MS: Ultimate 3000 RSLCnano and Q-Exactive HF respectively, both Thermo Fisher Scientific. Peptides were sampled on a 300 μm x 5 mm PepMap C18 precolumn (Thermo Fisher Scientific) and separated on a 75 μm x 250 mm C18 column (Reprosil-Pur 120 C18-AQ, 1.9 μm, Dr. Maisch HPLC GmbH). The nano-LC method consisted of a 60-min multi-linear gradient ranging from 5 to 42% acetonitrile in 0.1% formic acid at a flow rate of 300 nL/min. The spray voltage was set at 2 kV and the temperature of the heated capillary was adjusted to 270 °C. Survey full-scan MS spectra (m/z = 400–1600) were acquired with a resolution of 60 000 (m/z 200) after accumulation of 3 x 10^6^ ions (maximum injection time 55 ms), with lock mass activated. The 15 most intense ions were fragmented by high-energy collisional dissociation (HCD) after the accumulation of 10^5^ ions (maximum injection time: 120 ms). MS/MS resolution was set to 30 000 (m/z 200). MS and MS/MS data were acquired in data-dependent acquisition mode (DDA) using Q Exactive HF Tune software, v. 2.8.1 (Thermo Scientific).

1. *ELISA procedure:*

ELISA kits for ALS (Mediagnost, Kusterdingen, Germany) and Gal-3BP (CUSABIO, Houston, TX, USA) were used to quantify ALS and Gal-3BP in plasma for the evaluation (Grenoble cohort) and validation (Angers cohort) studies. Tests were performed according to the manufacturer’s instructions. Samples were diluted at 1:150 (10/1490, v/v sample/sample buffer) for ALS and 1:100 (10/40 – 15/285, v/v sample/sample buffer) for Gal-3BP. Briefly, 50 µL (for ALS assay) and 100 µL (for Gal-3BP assay) of each sample were added to pre-coated plates in duplicate and incubated for 2 h. After washing, enzyme conjugate was added to each well and incubated according to instructions. Another washing cycle was completed before adding substrate solution to each well and incubating in the dark. Finally, the stop solution was added to each well before measuring the optical density at 450 nm. Values were corrected with a reading at 540 nm. The standard curve was created using reference samples provided by manufacturers, ranging from 0 ng/mL to 200 ng/mL for the ALS assay and from 1.56 ng/mL to 100 ng/mL for the Gal-3BP assay. Interpolation of sample concentrations was achieved by comparison with the standard curve fitted with an asymmetrical sigmoidal (5PL) equation, and by multiplying by dilution factor. Samples 14 and 15 for Gal-3BP (Grenoble), 219 for ALS (Grenoble), 426 for Gal-3BP (Angers) and 471 for ALS (Angers) were considered as outliers in the ELISA results.

1. *CIs for ROC curves and AUCs:*

95% CIs around ROC curves were computed over 2000 stratified bootstrapped replicates of the cohort, using the [pROC](https://cran.r-project.org/web/packages/pROC/pROC.pdf) R package, as described in Carpenter and Bithell (2000) ^4^ sections 2.1 and 3.3. 95% CIs for AUCs were also computed using pROC, but applying the DeLong methodology ^5^, as it is an asymptotically exact method.

1. *Model {ALS, Gal-3BP}*

**Table 2: Weightings for the {ALS, Gal-3BP} model fitted to data from the Grenoble cohort for the logistic regression model.**

| Variable | Weighting |
| --- | --- |
| ALS (ng/L) | -0.0002883 |
| Gal-3BP (ng/L) | 0.001623 |
| Intercept | -0.2957 |

*References:*

1. Design and validation of a histological scoring system for nonalcoholic fatty liver disease - Kleiner - 2005 - Hepatology - Wiley Online Library. https://aasldpubs.onlinelibrary.wiley.com/doi/full/10.1002/hep.20701.

2. Boursier, J. *et al.* Screening for therapeutic trials and treatment indication in clinical practice: MACK-3, a new blood test for the diagnosis of fibrotic NASH. *Alimentary Pharmacology & Therapeutics* **47**, 1387–1396 (2018).

3. Bedossa, P. & Consortium, the F. P. Utility and appropriateness of the fatty liver inhibition of progression (FLIP) algorithm and steatosis, activity, and fibrosis (SAF) score in the evaluation of biopsies of nonalcoholic fatty liver disease. *Hepatology* **60**, 565–575 (2014).

4. Carpenter, J. & Bithell, J. Bootstrap confidence intervals: when, which, what? A practical guide for medical statisticians. *Stat. Med.* (2000).

5. DeLong, E. R., DeLong, D. M. & Clarke-Pearson, D. L. Comparing the areas under two or more correlated receiver operating characteristic curves: a nonparametric approach. *Biometrics* **44**, 837–845 (1988).
